# Control of Ni/β-Ga_2_O_3_ Vertical Schottky Diode Output Parameters at Forward Bias by Insertion of a Graphene Layer

**DOI:** 10.3390/nano12050827

**Published:** 2022-03-01

**Authors:** Madani Labed, Nouredine Sengouga, You Seung Rim

**Affiliations:** 1 Laboratory of Semiconducting and Metallic Materials (LMSM), University of Biskra, Biskra 07000, Algeria; madani.labed@univ-biskra.dz (M.L.); n.sengouga@univ-biskra.dz (N.S.); 2 Department of Intelligent Mechatronics Engineering and Convergence Engineering for Intelligent Drone, Sejong University, Seoul 05006, Korea

**Keywords:** SBD, β-Ga_2_O_3_, electron-beam evaporation, interfacial traps, graphene, workfunction, bandgap

## Abstract

Controlling the Schottky barrier height ($\phi_{B}$) and other parameters of Schottky barrier diodes (SBD) is critical for many applications. In this work, the effect of inserting a graphene interfacial monolayer between a Ni Schottky metal and a β-${\mathrm{Ga}}_{2}O_{3}$ semiconductor was investigated using numerical simulation. We confirmed that the simulation-based on Ni workfunction, interfacial trap concentration, and surface electron affinity was well-matched with the actual device characterization. Insertion of the graphene layer achieved a remarkable decrease in the barrier height ($\phi_{B}$), from 1.32 to 0.43 eV, and in the series resistance ($R_{S}$), from 60.3 to 2.90$\;mΩ.{\mathrm{cm}}^{2}$. However, the saturation current ($J_{S}$) increased from $1.26\times 10^{-11}\;$ to $8.3\times 10^{-7}$(A/cm^2^). The effects of a graphene bandgap and workfunction were studied. With an increase in the graphene workfunction and bandgap, the Schottky barrier height and series resistance increased and the saturation current decreased. This behavior was related to the tunneling rate variations in the graphene layer. Therefore, control of Schottky barrier diode output parameters was achieved by monitoring the tunneling rate in the graphene layer (through the control of the bandgap) and by controlling the Schottky barrier height according to the Schottky–Mott role (through the control of the workfunction). Furthermore, a zero-bandgap and low-workfunction graphene layer behaves as an ohmic contact, which is in agreement with published results.

## 1. Introduction

Gallium oxide (Ga_2_O_3_) is a new oxide semiconductor material with a long and rich history [1,2]. Pioneer studies were performed in the 1960s but were almost forgotten for about three decades. However, in the last two decades, its ultra-wide bandgap (UWBG) of ~4.8 eV, high breakdown electric field of ~8 MV/cm, and high saturation velocity of $1\times 10^{7}\mathrm{cm}/s$ have brought Ga_2_O_3_ to the fore again [3,4,5]. Ga_2_O_3_ has six polymorphs: α, β, γ, δ, ε and k, with β-${\mathrm{Ga}}_{2}O_{3}$ being the most stable [4]. Furthermore, it can be grown directly from the melt at a low cost and allows for large-scale production compared with GaN, InGaN, and SiC [1,2,4]. However, this material has a problem with developing a stable p-type [2,5,6,7]. As a result, its use in bipolar devices is limited to a heterojunction with other p-type materials such as NiO [8,9] and Cu_2_O [10]. β-Ga_2_O_3_ is therefore mainly used in unipolar devices (SBD [4,11], MOSFET [12], Thin-Film Transistor (TFT) [13], and field emission [14] devices). The SBD device based on a UWBG semiconductor is of great interest and, aimed to improve the thermal stability and decrease the series resistance ($R_{s}$), ideality factor (n), and leakage current. A low threshold voltage ($V_{Th}$) is preferred in order to minimize heating during prolonged device operation [15]. In addition to the above-mentioned characteristics, researchers aimed to develop SBDs with a controllable Schottky barrier height ($\phi_{B}$), with the aim of developing SBD-based switching devices for special applications. According to the Schottky–Mott relation [16], the linear relation between the metal workfunction ($\phi_{M}$) and $\phi_{B}$ can be set to ($\frac{\partial \phi_{B}}{\partial \phi_{M}}=1$). However, in most materials, this relation is not realistic due to unexpected effects such as the formation of interface states or an interface dipole [16]. Farzana et al. [17] studied the influence of the choice of metal in a (010) β-${\mathrm{Ga}}_{2}O_{3}$ SBD. They used different metals, namely Pd, Ni, Pt, and Au, with a workfunction of 5.20, 5.25, 5.65, and 5.30 eV, respectively, and obtained for each metal an $\phi_{B}$ of 1.27, 1.54, 1.58, and 1.71 eV, respectively. After linking the metal workfunction values and the obtained $\phi_{B}$, a modified Schottky–Mott relation for Au was obtained. Yao et al. [18] used W, Cu, Ni, Ir, and Pt with workfunctions of 4.55, 4.65, 5.15, 5.27, and 5.65 eV, respectively, and the corresponding $\phi_{B}$ values from the capacitance voltage were 1.94, 1.61, 1.61, 2.3, and 1.9 eV, respectively. It was also observed that $\phi_{B}$ does not show a universal trend with the metal workfunction, indicating that surface/interface states can play a very important role in determining an effective $\phi_{B}$ value. This is due to defects and the crystal orientation, crystal quality, and passivation with different types of surface treatments or metal deposition techniques. Among the different solutions that have been proposed to address these issues is depositing a layer with known properties between the metal and the β-${\mathrm{Ga}}_{2}O_{3}$ that can improve the SBD’s performance and allow for better $\;\phi_{B}$ control. Bhattacharyya et al. [19] studied the modulation and enhancement of $\phi_{B}\;$ for different β-${\mathrm{Ga}}_{2}O_{3}$ orientations and metals when an ultra-thin ${\mathrm{SiO}}_{2}$ layer is inserted at the metal–β-${\mathrm{Ga}}_{2}O_{3}$ interface. Harada et al. [15] reported systematic variations in $\phi_{B}$ in a metal/${\mathrm{PdCoO}}_{2}/$β-${\mathrm{Ga}}_{2}O_{3}$ SBD while increasing the thickness of the inserted ${\mathrm{PdCoO}}_{2}$ layer; the obtained results demonstrated good control of $\phi_{B}$ in a wide range (0.7 to 1.9 eV).

In this article, a new approach is proposed to improve and control the Schottky barrier height and other parameters of Ni/β-${\mathrm{Ga}}_{2}O_{3}$ by inserting a graphene layer at the interface between the Ni and the β-${\mathrm{Ga}}_{2}O_{3}$ so that the outputs of the SBD are controlled by the tunneling rate through the graphene layer (by tuning the graphene bandgap) and by the barrier between the graphene and the β-${\mathrm{Ga}}_{2}O_{3}$ (by tuning the graphene workfunction).

## 2. Experimental

**Figure 1:**
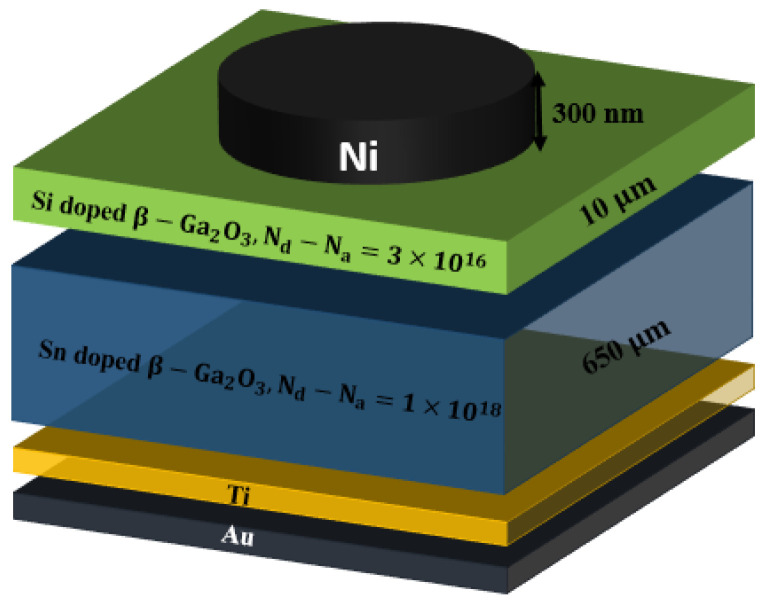
A schematic of the structure of the Ni/β-Ga_2_O_3_ SBD studied in this work.

A β-Ga_2_O_3_ Schottky barrier diode (SBD) based on an epitaxial Si-doped (001) β-Ga_2_O_3_ drift layer was deposited on a Sn-doped (001) β-Ga_2_O_3_ substrate by halide vapor-phase epitaxy (HVPE). A nickel film was deposited on the drift layer using an electron-beam evaporation method followed by annealing at 400 °C. A schematic illustration of the SBD structure is shown in Figure 1. The thickness of the nickel, Si-doped β-Ga_2_O_3_, and Sn-doped β-Ga_2_O_3_ was 0.3, 10, and 650 μm, respectively, while the doping was $1\times 10^{18}$ and $3\times 10^{16}{\;\mathrm{cm}}^{-3}$, respectively. For more details, see our previous publication [20].

## 3. Simulation Methodology

SILVACO ATLAS TCAD software (License Number 14556 License Period 18-3-21 to 17-3-22, Biskra Université Laboratory of Metallic and Semiconducting Materials FSESNV, Biskra, Algeria) was used in this simulation to solve the basic drift-diffusion semiconductor equations, which are the Poisson and continuity equations.

The Poisson equation is given by [2,21]:

(1) $$div\left(\varepsilon \nabla \psi \right)=-q\left(p-n+N_{d}\pm N_{t}^{\pm }\right)$$

where ψ is the electrostatic potential, ε is the permittivity, p and n are the free holes and electron concentrations, respectively, and $N_{t}^{\pm }$ is the trap’s ionized density $\left({\mathrm{cm}}^{-3}\right)$.

The continuity equations for electrons and holes are defined in steady states by [2,21]:

(2) $$0=\frac{1}{q}div\vec{J_{p}}+G_{n}-R_{n}$$

(3) $$0=-\frac{1}{q}div\vec{J_{p}}+G_{p}-R_{p}$$

where $G_{n}$ and $G_{p}$ are the generation rates for electrons and holes, respectively, $R_{n}$ and $R_{p}$ are the recombination rates for electrons and holes, respectively, $\vec{J_{n}}$ and $\vec{J_{p}}$ are the electron and hole current densities, respectively, which are given in terms of the quasi-Fermi level ($\phi_{n}$ and $\phi_{p}$) and mobility ($\mu_{n}$ and $\mu_{p}$) as [2,21]:

(4) $$\vec{J_{n}}=-q\mu_{n}n\nabla \phi_{n}$$

(5) $$\vec{J_{p}}=-q\mu_{p}p\nabla \phi_{p}$$

Traps are represented by their ionized density $\;N_{t}^{\pm }$. The sign ± depends on whether the trap is an acceptor or a donor so that $N_{t}^{+}=fN_{t}$ and $N_{t}^{-}=\left(1-f\right)N_{t}$, where f is the occupancy function given by $\;f=\frac{\sigma_{n}n+\sigma_{p}p}{\sigma_{n}\left(n+n_{t}\right)+\sigma_{p}\left(p+p_{t}\right)}$ and $\sigma_{n\left(p\right)}$ is the trap capture cross-section for electrons (holes). Furthermore, the recombination rate is related to traps through the well-known SRH formula $R_{n,p}=\frac{pn-n_{i}^{2}}{\tau_{0n}\left(p+p_{t}\right)+\tau_{0p}\left(n+n_{t}\right)}$ with $n_{t}=n_{i}exp\left(-\left(E_{i}-E_{t}\right)/kT\right)$ and $p_{t}=n_{i}exp\left(-\left(E_{t}-E_{i}\right)/kT\right)$. $\tau_{0n}$ and $\tau_{0p}$ are the minority carrier lifetimes, which are also related to traps through $\tau_{0n\left(p\right)}=\frac{1}{v_{thn\left(p\right)}\sigma_{n\left(p\right)}N_{t}}$, where $v_{thn\left(p\right)}$ is the thermal velocity of electrons (holes). The external generation rate $G_{n,p}$ is neglected since the forward bias is within low injection levels [2]. The tunneling mechanism through graphene, which has an important effect [22,23], has to be considered together with thermionic emissions, Shockley–Read–Hall and Auger recombination, Klassen’s concentration and mobility-dependent temperature, and a reduction in the image force in the simulation. For the graphene layer’s simulation, we considered this layer to be an ultra-thin (0.34 nm thickness) semiconductor with high mobilities, a low tunneling mass, and a low bandgap (0–0.45 eV). The dominant transport mechanism of electrons from Ni to graphene is a tunneling mechanism. Tunnelling was considered by using the Universal Schottky Tunnelling (UST) model and the tunnelling current is given by [7]:

(6) $$J_{T}=\frac{A^{*}T_{L}}{K_{B}}\overset{\infty }{\underset{\epsilon }{\int }}\Gamma \left(E^{\prime }\right)\ln\left(\frac{1+F_{s}\left(E^{\prime }\right)}{1+F_{m}\left(E^{\prime }\right)}\right)dE^{\prime }$$

where $A^{*}$, $T_{L}$, $K_{B}$, *ϵ*, $F_{s}\left(E^{\prime }\right)$, and $F_{m}\left(E^{\prime }\right)$ are the effective Richardson’s coefficient (41.11 ${\mathrm{Acm}}^{-2}K^{-2}$ for β-Ga_2_O_3_ [2]), the lattice temperature, the Boltzmann constant, the electron energy, and the Maxwell–Boltzmann distribution in the semiconductor and metal, respectively, and $\Gamma \left(E^{\prime }\right)$ is the tunnelling probability given by [7]:

(7) $$\Gamma \left(\epsilon \right)=\exp\left[-2\frac{\sqrt{2m^{*}}}{\hbar }\int_{x1}^{x2}\sqrt{E_{c}\left(x\right)-\epsilon }dx\right]$$

where $E_{c}\left(x\right)$, ($x_{1},\;x_{2})$, and $m^{*}$ are the potential energy distribution of the Schottky barrier diode, the classical turning points, and the tunnelling mass in graphene ($m^{*\;}$= 0.012m_0_ where m_0_ is the free electron mass [24]), respectively. In addition, the thermionic emission plays an important role in this type of device. The properties and traps related to each layer are presented in Table 1 and Table 2, respectively.

## 4. Results

**Figure 2:**
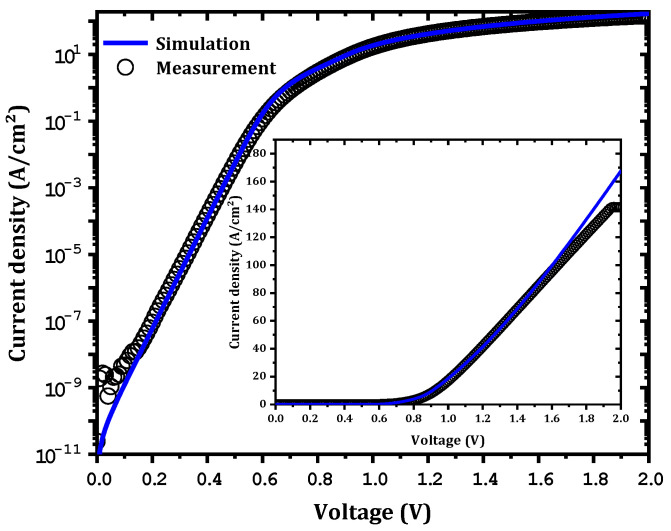
Comparison between the simulation data and experimental measurements. The inset is a linear representation of the J-V characteristics.

As presented in Figure 2, a good comparison between the simulation data and the experimental measurements was obtained. The extracted Ni workfunction, interfacial trap concentrations, and surface electron affinity from SILVACO ATLAS software were 5 eV, $8\times 10^{15}\;{\mathrm{cm}}^{-3}$ for all traps ($E_{2}$ ($E_{c}-0.75$), $E_{2}^{*}$ ($E_{c}-0.72$), and $E_{3}$ ($E_{c}-1.05$)), and 3.89 eV, respectively.

### 4.1. Effect of Insertion of a Graphene Layer

**Figure 3:**
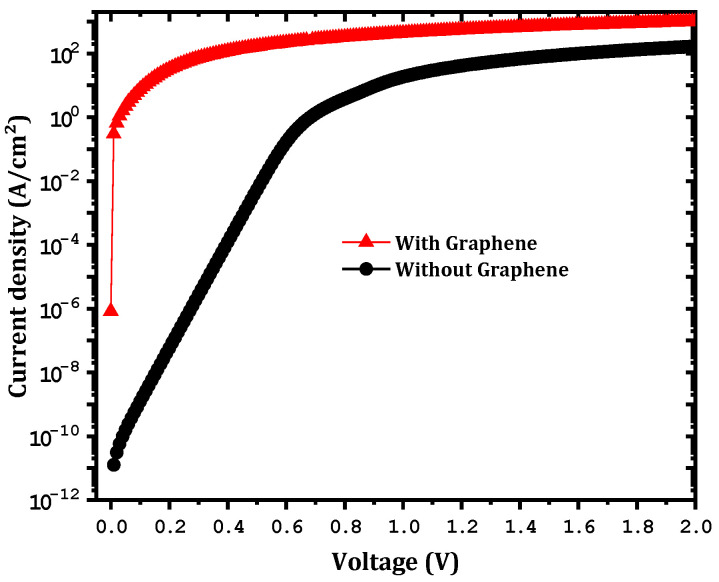
The effect of the insertion of a graphene layer on the forward current.

**Figure 4:**
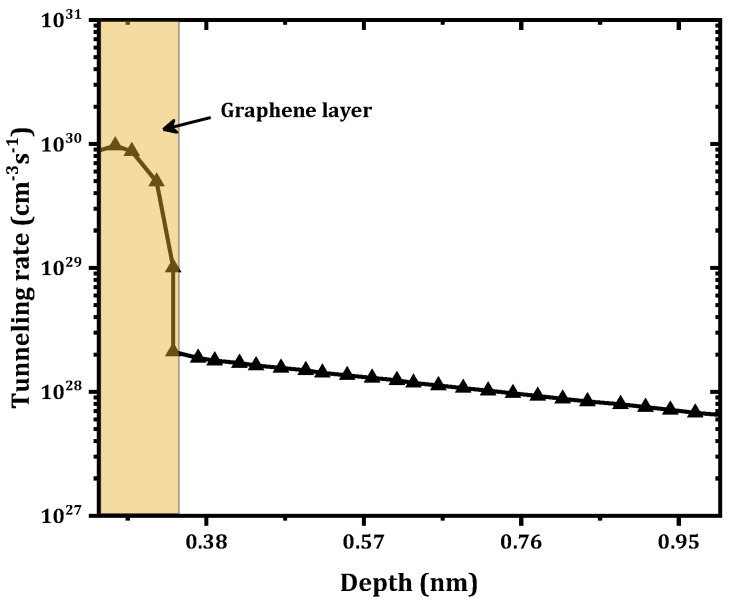
Variation in the tunneling rate when a graphene monolayer was inserted under 2 V of forward voltage.

The main goal of the present work was to study the effect of inserting a graphene monolayer between the Ni and the ${\mathrm{Ga}}_{2}O_{3}$. This monolayer has the potential ability to come into contact with the ${\mathrm{Ga}}_{2}O_{3}$ at a low Schottky barrier height. As shown in Figure 3, a strong effect on the forward current and a reduction in the Schottky barrier height from 1.32 to 0.43 eV were obtained. These results are in agreement with those of Yuan et al. [27], where a very low $\phi_{B}$ was obtained. The results are also in agreement with those of Zhong et al. [28], who found a decrease in $\phi_{B}$ when a graphene layer was inserted into a GaN SBD. According to Courtin et al. [22], a similar variation for a graphene–silicon interface was obtained. Inaba et al. [29] also found a very low $\phi_{B}$ at a CNT–SiC interface. In addition, a decrease in the series resistance ($R_{s}$) from 60.3 to 2.90 $mΩ{\;\mathrm{cm}}^{2}$ was obtained. However, an increase in the saturation current from $1.26\times 10^{-11}\;$ to $8.3\times 10^{-7}$(A/cm^2^) was observed after the insertion of this graphene layer. This decrease in $\phi_{B}$ and $R_{s}$ along with the increase in the saturation current when the graphene monolayer was inserted can be explained by the increase in the tunneling rate, especially at the interface between the Ni and the graphene as shown in Figure 4. Accordingly, for the electron transport from the Ni to the graphene layer (assuming sufficient energy to overcome the barrier), the thermionic emission dominated. Otherwise, when the electron energy was lower than the barrier energy, tunneling played an important role, especially at the Ni–graphene interface.

With the graphene layer, the tunneling mechanism increased the density of the extracted free electrons from the Ni to the graphene and then to the β-${\mathrm{Ga}}_{2}O_{3}$ by the thermionic emission and tunneling. This led to a large decrease in $R_{s}$. In addition, electron tunneling through the formed barriers between Ni/graphene and graphene/β-${\mathrm{Ga}}_{2}O_{3}$ affected the SBD parameters.

### 4.2. Graphene Bandgap Effect

**Figure 5:**
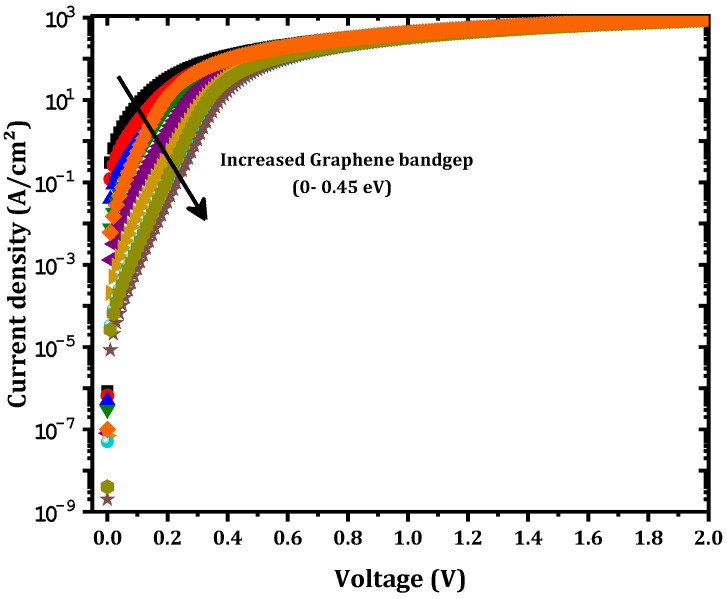
Output J-V variations with the graphene bandgap.

We demonstrated that a graphene monolayer can enhance the SBD outputs by increasing the tunneling rate. Experimentally, the graphene bandgap can be controlled by several methods. Takahashi et al. [30] found that the bandgap gradually increases with oxygen adsorption to as high as 0.45 eV upon exposure to 2000 L of oxygen. Additionally, the bandgap can be increased by atomic and molecular doping control, such as the simultaneous insertion of holes and electrons at hetero sites [31]. Altering the number of graphene layers is another way of tuning the graphene bandgap [32]. Bearing in mind these facts, the effect of the graphene bandgap was investigated and, as shown in Figure 5, as the graphene bandgap increased from 0 to 0.45 eV, the output current was affected.

**Figure 6:**
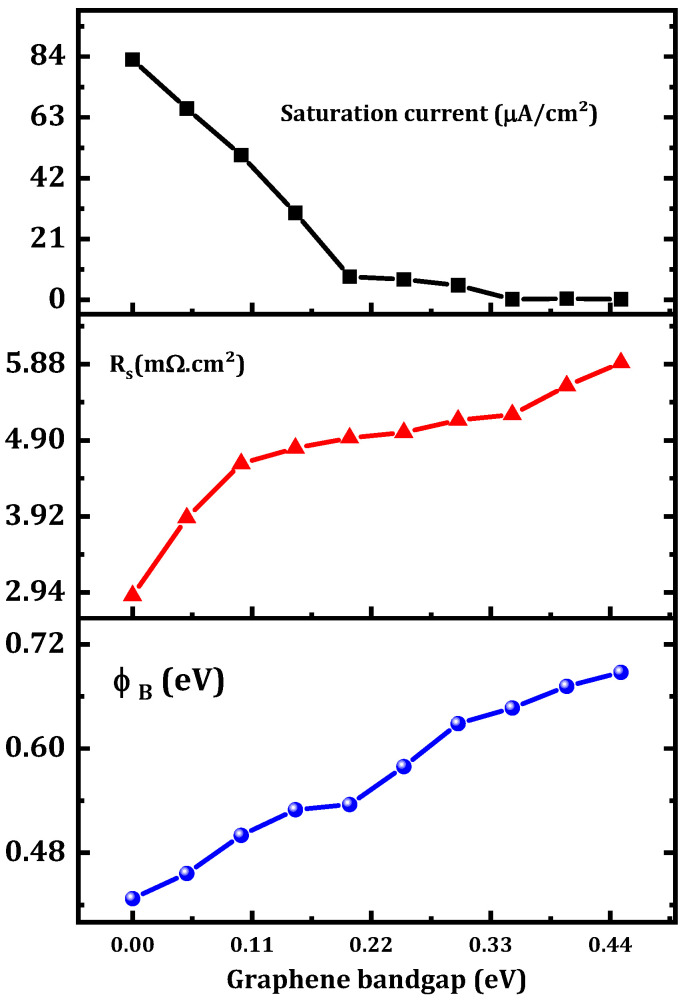
Variation in the output parameters of the proposed SBD versus the graphene bandgap.

**Figure 7:**
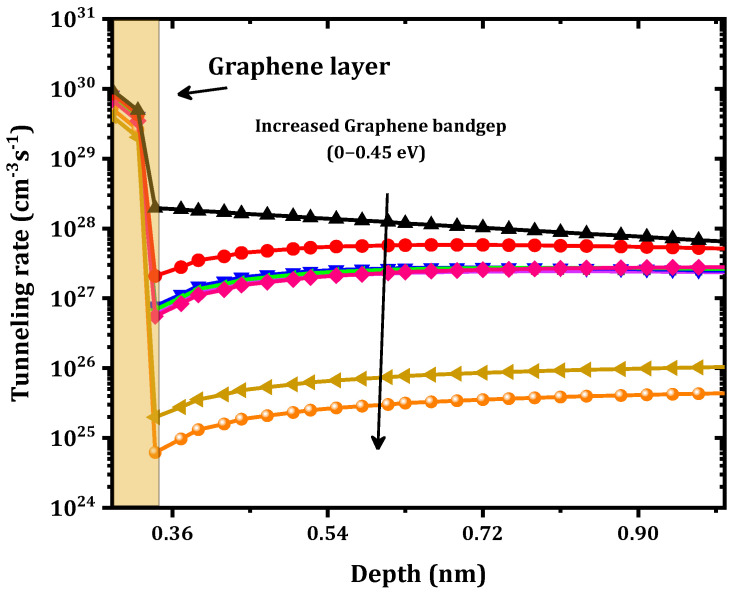
Variation in the tunneling rate versus the graphene bandgap under 2 V of forward voltage.

**Figure 8:**
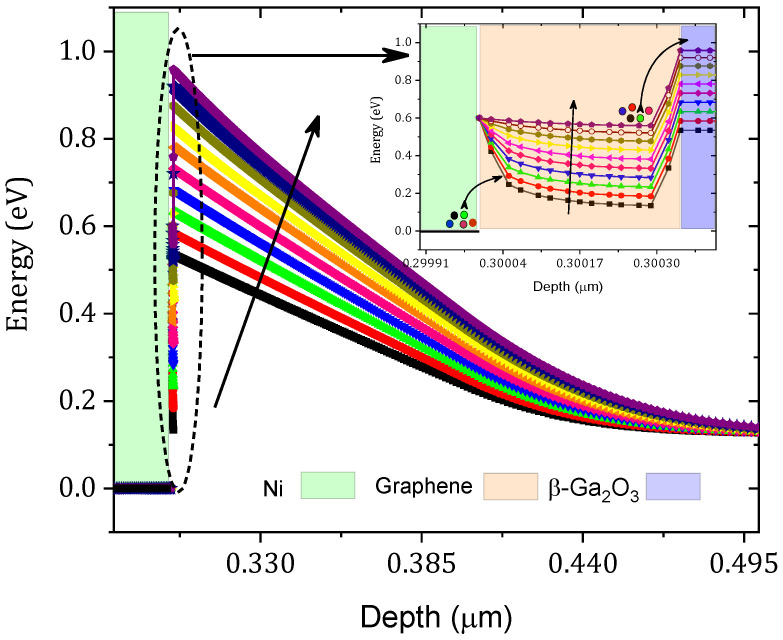
Equilibrium conduction band variation with the graphene workfunction. The inset is a zoomed Ni/graphene/ β-${\mathrm{Ga}}_{2}O_{3}$ interface conduction band.

The $\phi_{B}$ value increased from 0.43 eV to 0.69 eV and the series resistance increased from 2.90 to 5.90 mΩ cm^2^ as shown in Figure 6. This result can be interpreted as a decrease in the tunneling rate, as shown in Figure 7. This decrease in the tunneling rate is related to the increase in the potential energy distribution of the Schottky barrier diode as presented in Figure 8. A high tunneling rate was obtained in most cases at the Ni–graphene interface that was higher than that at the graphene–β-${\mathrm{Ga}}_{2}O_{3}$ interface. The obtained values demonstrate the possibility of tuning the Schottky barrier height of a Ni–β-${\mathrm{Ga}}_{2}O_{3}$ Schottky diode through the control of the tunneling rate in the graphene layer. Figure 8 shows the conduction band variation for the Ni/graphene/β-${\mathrm{Ga}}_{2}O_{3}$ SBD with an increasing graphene bandgap. The barrier between the Ni and the graphene increased and this affected the electron tunneling from the Ni to the graphene. In addition, a small increase in the barrier between the graphene and the β-${\mathrm{Ga}}_{2}O_{3}$ was observed and this led to an increase in $\phi_{B}$.

This decrease in the tunneling rate led to a decrease in the saturation current as presented in Figure 5 and Figure 6.

### 4.3. Graphene Workfunction Effect

**Figure 9:**
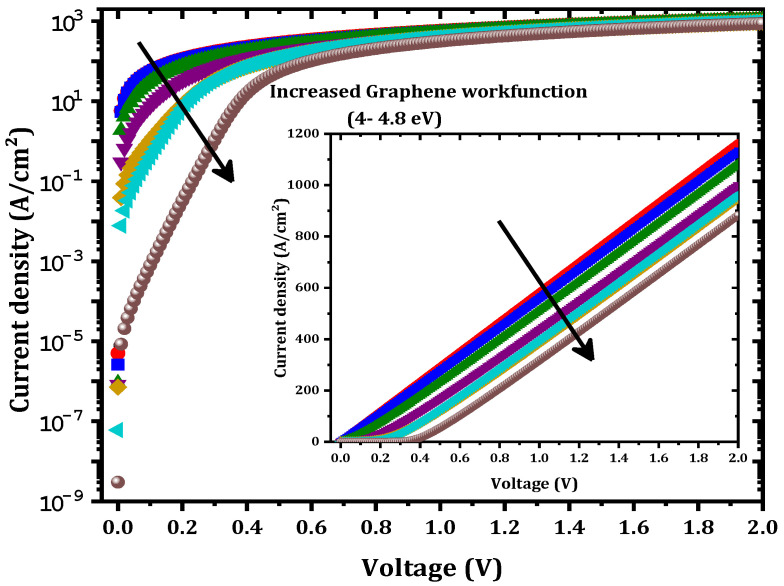
Output J-V variation versus the graphene workfunction. The inset is a linear representation of the J-V characteristics.

**Figure 10:**
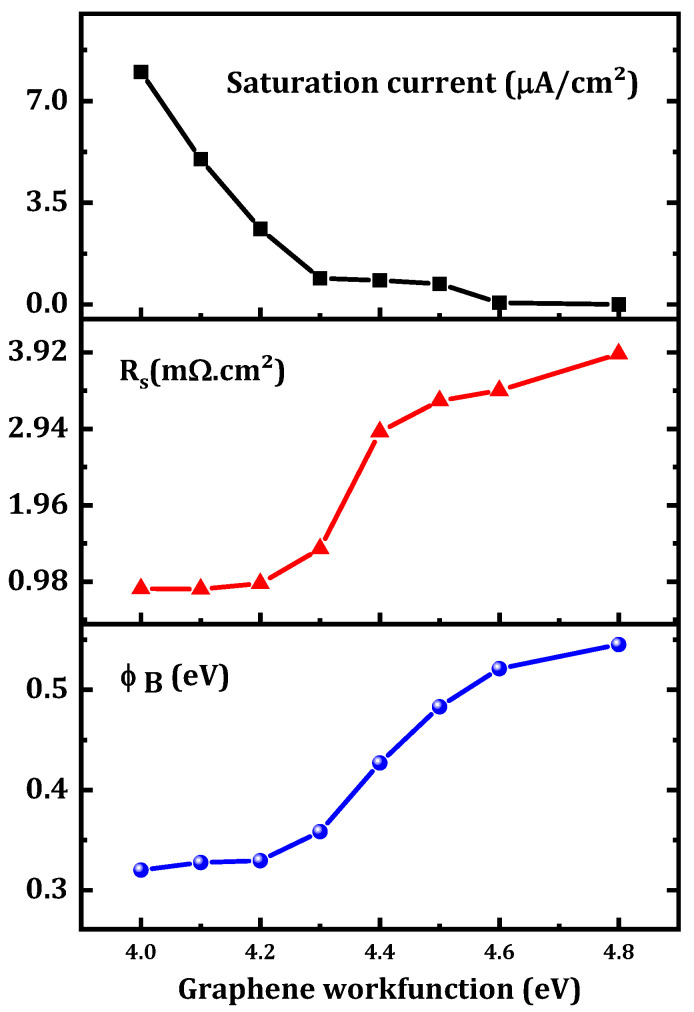
Variation in the output parameters of the proposed SBD versus the graphene workfunction.

The effect of the graphene workfunction on SBD parameters was investigated. In this study, a zero-bandgap graphene layer was considered. As the graphene workfunction increased from 4 to 4.8 eV, the output current was affected (Figure 9) and $\phi_{B}$ increased from 0.320 eV to 0.545 eV as presented in Figure 10. This increase in $\phi_{B}$ can be interpreted according to the simple Schottky–Mott model as the difference between the workfunction of graphene ($W_{G}$) and the affinity of β-${\mathrm{Ga}}_{2}O_{3}$($\chi_{{\mathrm{Ga}}_{2}O_{3}}$) [22]:

(8) $$\phi_{B}=W_{G}-\chi_{Ga_{2}O_{3}}$$

Furthermore, a decrease in the saturation current was obtained with the increase in the graphene workfunction.

**Figure 11:**
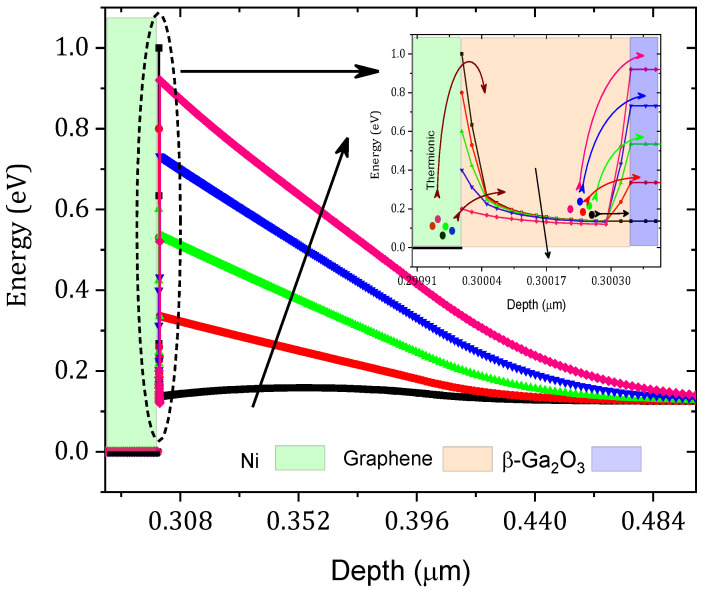
Variation in the equilibrium conduction band with the graphene workfunction. The inset is a zoomed Ni/graphene/β-${\mathrm{Ga}}_{2}O_{3}$ interface conduction band.

As the graphene workfunction increased, $R_{s}$ increased from 0.89 to 3.9 mΩ.cm^2^. This is related to the decrease in the tunneling rate in the graphene layer. Generally, controlling the graphene workfunction means controlling $\phi_{B}$ between the graphene and the β-$Ga_{2}O_{3}$ as presented in Figure 11. As the workfunction increases, the barrier between the graphene and the β-${\mathrm{Ga}}_{2}O_{3}$ increases while that between the Ni and the graphene decreases. This leads to a decrease in the number of electrons transported from the graphene to the β-${\mathrm{Ga}}_{2}O_{3}$ by thermionic emission. Therefore, an increase in $R_{s}$ and a decrease in the saturation current were obtained with an increase in the graphene workfunction. Experimentally and as described in [31,32], the graphene workfunction can be controlled by altering the number of graphene layers. The workfunction increased as well, reaching 4.8 eV. In addition, altering the graphene doping density is an essential method for controlling the graphene workfunction because doping the graphene layer changes the fermi-level of the graphene, which affects the electronic properties of the graphene and, among the properties, the workfunction [33,34]. In addition, a zero-bandgap and low-workfunction SBD behaves as an ohmic contact as shown in the inset of Figure 9 (the current has a linear variation versus the forward voltage). Figure 11 shows the equilibrium band diagram. For a low workfunction (4 eV), a very low barrier is formed between the graphene and the β-${\mathrm{Ga}}_{2}O_{3}$. It was therefore concluded that a graphene layer with a lower workfunction (4 eV) and a 0-eV bandgap transitioned from a Schottky contact to an ohmic contact. This result is in agreement with the simulation result obtained by Yuan et al. [27].

## 5. Conclusions

In summary, this study investigated the effect of a graphene layer on characteristics of a Ni/β-${\mathrm{Ga}}_{2}O_{3}$ SBD. Firstly, good agreement between simulation data and experimental measurements was obtained for the Ni/β-${\mathrm{Ga}}_{2}O_{3}$ SBD without a graphene layer with the consideration of a 5 eV Ni workfunction, an $8\times 10^{15}\;{\mathrm{cm}}^{-3}$ density for the $E_{2}$ ($E_{c}-0.75$), $E_{2}^{*}$ ($E_{c}-0.72$), and $E_{3}$ ($E_{c}-1.05$) interfacial traps, and a 3.89 eV surface electron affinity. Then, the effect of inserting a zero-bandgap graphene layer at the interface between the Ni and the β-${\mathrm{Ga}}_{2}O_{3}$ was studied. We observed a decrease in both $\phi_{B}$ and the series resistance $R_{S}$. However, the saturation current *n* increased. These effects were related to an increase in the tunneling rate. The graphene bandgap and workfunction were used to control the output parameters of the SBD. When the graphene bandgap increased, the Schottky barrier height and series resistance both increased. Similarly, $\phi_{B}$ and $R_{S}$ increased as well. In addition, with a lower graphene workfunction, Ni behaved as an ohmic contact with β-${\mathrm{Ga}}_{2}O_{3}$.

## Figures and Tables

**Table 1:** Properties of each layer of the studied SBD [2,25].

Parameters	Sn: β-Ga_2_O_3_	Si: β-Ga_2_O_3_	Graphene
Bandgap (eV)	4.8	4.8	0–0.45
Affinity (eV)	4	4	4–4.8
$\mathrm{Hole}\;\mathrm{mobility}\;({\mathrm{cm}}^{2\;}V^{-1}{\;s}^{-1})$	10	10	9000
$\mathrm{Electron}\mathrm{mobility}\;({\mathrm{cm}}^{2}{\;V}^{-1}{\;s}^{-1})$	172	300	9000
Relative permittivity	12.6	11	6.9
$N_{c}\;({\mathrm{cm}}^{-3})$	$3.7\times 10^{18}$	$3.7\times 10^{18}$	$1\times 10^{19}$
$N_{v}\;({\mathrm{cm}}^{-3})$	$5\times 10^{18}$	$5\times 10^{18}$	$1\times 10^{19}$
$N_{d}\;({\mathrm{cm}}^{-3})$	$1\times 10^{18}$	$3\times 10^{16}$	/
Thickness (µm)	650	10	0.34 × 10^−3^

**Table 2:** Traps related to β-Ga_2_O_3_ layers [2,4,26].

**Traps**	Trap Level $\left(E_{c}-E)\;(\mathrm{eV}\right)$	Trap Concentration $\left(cm^{-3}\right)$	$\mathrm{Capture}\;\mathrm{Cross-}\mathrm{Section}\;\sigma_{n}\;({\mathrm{cm}}^{2})$	$\sigma_{n}$ $/\sigma_{p}$
Sn-doped β-Ga_2_O_3_ Bulk layer	0.55 0.74 1.04	$3\times 10^{13}\;2\times 10^{16}\;4\times 10^{16}$	$2\times 10^{-14}\;2\times 10^{-14}\;2\times 10^{-14}$	100 100 10
Si-doped β-Ga_2_O_3_ thin layer	0.60 0.75 0.72 1.05	$3.6\times 10^{13}\;4.6\times 10^{13}\;4.6\times 10^{13}\;1.1\times 10^{14}$	$2\times 10^{-14}\;2\times 10^{-14}\;2\times 10^{-14}\;2\times 10^{-14}$	100 100 100 10
